# Inbreeding effects in a mixed-mating vine: effects of mating history, pollen competition and stress on the cost of inbreeding

**DOI:** 10.1093/aobpla/plv133

**Published:** 2015-11-17

**Authors:** Øystein H. Opedal, W. Scott Armbruster, Christophe Pélabon

**Affiliations:** 1 Centre for Biodiversity Dynamics, Department of Biology, Norwegian University of Science and Technology, 7491 Trondheim, Norway; 2 Department of Biology, Norwegian University of Science and Technology, 7491 Trondheim, Norway; 3 School of Biological Sciences, University of Portsmouth, King Henry Building, King Henry I Street, Portsmouth PO1 2DY, UK; 4 Institute of Arctic Biology, University of Alaska, Fairbanks, AK 99775, USA

**Keywords:** *Dalechampia scandens*, environmental stress, herkogamy, inbreeding depression, mixed mating systems, pollen competition

## Abstract

Inbreeding depression is assumed to be a central factor contributing to the stability of plant mating systems. Predicting the fitness consequence of inbreeding in natural populations is complicated, however, because it may be affected by the mating histories of populations generating variation in the amount of purging of deleterious alleles. Furthermore, the level of inbreeding depression may depend on environmental conditions and the intensity of pollen competition. In a greenhouse experiment comparing four populations of the neotropical vine *Dalechampia scandens* (Euphorbiaceae), we tested whether inbreeding depression for early-life fitness depended on the inferred mating history of each population, as indicated by genetically determined differences in herkogamy and autofertility rates. We also tested whether the intensity of pollen competition and the level of stress encountered by the seeds and seedlings affected the amount of inbreeding depression observed. Herkogamy was a good predictor of autofertility in each population. However, we found only limited evidence for inbreeding depression in any population, and inbreeding depression varied independently of the intensity of pollen competition and amount of stress encountered by the seeds and seedlings. Thus, the population's rate of autofertility did not predict the amount of inbreeding depression. Overall, we found no evidence supporting the expectations that more inbred populations experience less inbreeding depression, and that pollen competition reduces the cost of inbreeding. These results suggest that additional factors may be responsible for the maintenance of the mixed mating systems of *D. scandens* populations.

## Abstract (precis)

Reduced fitness of inbred offspring is assumed to be a main factor maintaining outcrossing in plants. If there is no cost of selfing, complete selfing is expected to evolve as a result of the increased number of alleles transmitted to inbred offspring. In *Dalechampia scandens*, and a number of other plant species, inbreeding depression appears not to be strong enough to explain the continued investment in outcrossing. In some of these species a stable mixed mating system might instead be maintained, for example, by pollinators generating positive correlations between female and male fitness components.

## Introduction

Despite more than a century of research into the causes of variation and evolution of plant mating systems, many questions remain unanswered (Karron *et al*. 2012). Early models suggested that self-fertilization (selfing) and outcrossing could be considered as alternative stable strategies and mixed mating a transitional state between the two (Lande and Schemske 1985), yet more than 40 % of plant species exhibit mixed mating systems (Goodwillie *et al*. 2005). Accordingly, attention has shifted towards identifying genetic and ecological factors that contribute to the stability of mixed mating systems (e.g. Cheptou and Mathias 2001; Johnston *et al*. 2009; Winn *et al*. 2011; see Goodwillie *et al*. 2005 for a review).

While selfing has obvious benefits in terms of genetic contribution to offspring (automatic selection; Fisher 1941), and reproductive assurance when outcross pollen is limited (e.g. Kalisz and Vogler 2003, see Busch and Delph 2012 for a review of both hypotheses), there are also several costs associated with selfing. Inbreeding depression, the reduced fitness of inbred compared with outcrossed offspring, reduces the fitness advantage of selfing and is expected to be the main factor favouring outcrossing (Lloyd 1979; Charlesworth and Charlesworth 1987, but see Johnston *et al*. 2009). Under the partial dominance hypothesis, inbreeding depression is assumed to be caused mainly by the expression of deleterious recessive alleles in inbred offspring (Charlesworth and Charlesworth 1987). However, the level of inbreeding depression can co-evolve with the mating system, therefore complicating the prediction of its selective effect (Lande and Schemske 1985; Charlesworth *et al*. 1990). Indeed, theory predicts that repeated episodes of inbreeding should purge the population of recessive deleterious alleles and restore the fitness of inbred offspring (e.g. Barrett and Charlesworth 1991; Husband and Schemske 1996; Byers and Waller 1999, but see Winn *et al*. 2011). The fitness costs of inbreeding are, therefore, expected to vary with the history of inbreeding in a population, and this variation might in turn influence the stability of mixed mating systems.

The evolution of plant mating systems is also expected to affect floral morphology and development (Barrett 2003; Harder and Johnson 2009; Goodwillie *et al*. 2010). Selection for outcrossing in self-compatible, animal-pollinated species generally favours the separation of male and female functions in time (dichogamy; Lloyd and Webb 1986) and/or space (herkogamy; Webb and Lloyd 1986). Genetic variation in such ‘mating-system traits’ among species, populations or even individuals is assumed to reflect variation in their mating histories. For example, variation in herkogamy has been shown to be associated with variation in autofertility (seed production in the absence of pollinators, e.g. Armbruster 1988; Moeller 2006; Brys *et al*. 2013) and outcrossing rate (e.g. Holtsford and Ellstrand 1992; Karron *et al*. 1997; Brunet and Eckert 1998; Motten and Stone 2000; Takebayashi *et al*. 2006; Herlihy and Eckert 2007). Hence, it is likely that populations with lower average herkogamy experience more selfing and purging of their genetic load, and should therefore suffer less from inbreeding depression than populations with greater herkogamy and outcrossing (e.g. Carr *et al*. 1997; Takebayashi and Delph 2000; Stone and Motten 2002; Goodwillie and Knight 2006; Dart and Eckert 2013).

Studies testing the purging hypothesis by comparing species or populations with different mating histories have yielded inconsistent results (Winn *et al*. 2011), and several authors have suggested that additional factors influence the severity of inbreeding depression. First, inbreeding depression has been shown to often be more severe in more stressful environments (Armbruster and Reed 2005; Cheptou and Donohue 2011; Fox and Reed 2011). Second, some studies have suggested that the cost of inbreeding might be reduced if self-pollen is allowed to compete for fertilization of ovules (Armbruster and Rogers 2004; Lankinen and Armbruster 2007). Because pollen is haploid, many deleterious recessive alleles otherwise hidden from selection in the diploid phase should be exposed to selection during the growth of the pollen tube (Mulcahy 1979; Mulcahy and Mulcahy 1987; Charlesworth and Charlesworth 1992; Armbruster and Rogers 2004). Two studies have found some evidence for the hypothesis that competition among self-pollen reduces the cost of inbreeding. Armbruster and Rogers (2004) observed that seeds resulting from self-fertilization with intense pollen competition in a population of *Dalechampia scandens* were 16 % heavier than seeds produced after less intense pollen competition. In *Collinsia heterophylla*, intense pollen competition tended to decrease inbreeding depression revealed in several components of fitness (Lankinen and Armbruster 2007). A third and hitherto untested prediction of the hypothesis that pollen competition reduces the cost of inbreeding is that the effect should be stronger in more outcrossing populations and species, due to the higher genetic load expected in such populations and species (Lankinen and Armbruster 2007).

These considerations show that understanding the role of inbreeding depression in the maintenance of mixed mating systems in plant populations requires carefully designed experiments assessing the joint effects of several factors. The goal of the present study was to assess the interrelated effects of mating history (as indicated by variation in floral traits and autofertility rates), intensity of pollen competition and amount of environmental stress on the expression of early-life inbreeding depression in a mixed-mating species. In a greenhouse experiment comparing four populations of *D. scandens*, we predicted that populations with less herkogamy would exhibit greater autofertility rates and experience less inbreeding depression than populations with greater herkogamy. By manipulating the intensity of pollen competition at constant pollen load (by varying pollen dispersion on the stigma), we also tested whether inbreeding depression was reduced by intense pollen competition, and whether this effect varied among populations. Finally, we compared levels of inbreeding depression, as reflected in germination, seedling survival and seedling growth, under stressful vs. benign environmental conditions.

## Methods

### Study species and populations

*Dalechampia scandens* (Euphorbiaceae) is a self-compatible vine with a mixed mating system, widely distributed in the Neotropics from Mexico to Argentina (Armbruster 1985). Male and female unisexual flowers are aggregated into bisexual blossom inflorescences, comprising a cluster of three pistillate flowers situated below a cluster of 10 staminate flowers and subtended by two involucral bracts (Webster and Webster 1972). Each ovary contains three ovules, so that blossoms can produce a maximum of nine seeds. The blossoms are functionally protogynous, with a female phase (stigmas receptive, anthers not dehisced) followed by a bisexual phase. Auto-fertilization can occur during the bisexual phase, although the rate at which it occurs seems to depend on the physical distance separating anthers and stigmas (i.e. herkogamy; Armbruster 1988, and see Results). The degree of herkogamy differs among populations and depends, at least partly, on the geometry of the blossom (the relative orientation of male and female flowers; Armbruster *et al*. 2009). Furthermore, herkogamy is a highly evolvable trait, as indicated by the large additive genetic variance harboured in one of the study populations (Hansen *et al*. 2003). *Dalechampia scandens*, thus, represents an interesting system for studying the consequences of variation in floral traits (and presumed mating history) for the expression of inbreeding depression and the evolution and stability of mixed mating systems.

The *D. scandens* species complex was recently shown to comprise at least two cryptic species differing in floral morphology (Bolstad *et al*. 2014). In the present study, we chose to include only populations from the large-glanded species because this species exhibits a greater range of herkogamy among populations, suggesting greater variation in their dependence on selfing, and because genetic analyses (M. Falahati-Anbaran, G. H. Bolstad, C. Pélabon, Ø. H. Opedal and W. S. Armbruster, unpubl. res.) have suggested that the small-glanded species is polyploid, thus complicating predictions of the effect of purging. The four study populations originate from the Yucatan Peninsula in Mexico and differ greatly in average herkogamy (Table 1). Differences in herkogamy can result from both size and shape differences among blossoms (Armbruster *et al*. 2009). The population from La Mancha (LM) has anthers and stigmas diverging in more or less the same dimension away from the resin gland (Armbruster *et al*. 2009). Average herkogamy in this population is just over 1 mm, suggesting frequent self-pollination. Indeed, a phenotypic-selection study on this population detected selection for selfing (reduced anther–stigma distance (ASD)), and exclusion of pollinators only marginally decreased blossom seed set in the field (Pérez-Barrales *et al*. 2013). In contrast, the populations from Tulum (T), Puerto Morelos (PM) and Ciudad del Carmen (CC) exhibit blossom geometries associated with greater herkogamy. Hence, we assumed that they rely, in the wild, more on pollinator-mediated cross-pollination.

**Table 1.** Location and summary statistics of study populations used to investigate inbreeding effects in *D. scandens*. Herkogamy and dichogamy were measured in the greenhouse on 30 plants from each population.

Population	Coordinates	Herkogamy ± SE (mm)	Dichogamy ± SE (days)
LM	19°37′15″N, 96°28′09″W	1.29 ± 0.15	2.33 ± 0.07
PM	20°51′11″N, 86°53′43″W	2.73 ± 0.14	2.13 ± 0.10
CC	18°56′29″N, 91°18′01″W	2.83 ± 0.18	1.98 ± 0.14
T	20°12′26″N, 87°27′04″W	3.17 ± 0.12	2.43 ± 0.10

Like most *Dalechampia* species, the stigmas of *D. scandens* extend from the tip down the lateral surface of the styles (Armbruster *et al*. 1995). To reach the ovaries, pollen grains landing on the lateral surface must grow first to the stylar tip (i.e. away from the ovule), and then turn 180° to grow down the centre of the style towards the ovules (Armbruster *et al*. 1995). Hence, pollen grains landing on or near the stylar tip will have a competitive advantage over those landing further down the lateral surface of the style, and the intensity of pollen competition will, therefore, be affected by the positional variance of the pollen load (Armbruster and Rogers 2004). If most pollen arrives simultaneously on the tip of the stigma, the competition will be ‘fair’, and genetically superior gametophyte fathers will be expected to fertilize the ovules. If, on the other hand, pollen genotypes are randomly dispersed along the lateral stigmatic surface, pollen of lower quality might fertilize ovules by chance, and genetic variance in pollen-tube growth rates might be preserved in the population (Armbruster *et al*. 1995).

### Experimental design

The experimental populations were grown in November and December 2013 from seeds obtained from random within-population crosses of greenhouse plants grown from field-collected seeds. Hence, the plants used in the experiment represented the second greenhouse generation, except for the T population, which was the fifth greenhouse generation. Therefore, effects of maternal environments should be negligible, and any phenotypic differences among population observed in the greenhouse were largely genetically based.

Thirty plants from each population were used in the experiment. Using each maternal plant as an experimental block, we applied the following pollination treatments to randomly chosen blossoms of each plant:

1. *Inbred with intense pollen competition*. Emasculated blossoms were hand-pollinated with pollen from a haphazardly chosen blossom on the same plant (i.e. geitonogamous selfing). Pollen was deposited in a dense cluster on the tip of the stigma (low positional variance) to induce intense pollen competition (Mulcahy and Mulcahy 1975; Pélabon *et al*. 2016).
2. *Inbred with weak pollen competition*. Similar to (1), but with pollen highly dispersed along the stigmatic lateral surface (high positional variance), inducing weaker pollen competition.
3. *Outbred with intense pollen competition.* Emasculated blossoms were hand-pollinated with pollen from a haphazardly chosen blossom of an unrelated designated father plant (no shared grandparents). Pollen was deposited in a dense cluster on the tip of the stigma to induce intense pollen competition.
4. *Outbred with weak pollen competition*. Similar to (3), but with pollen highly dispersed along the stigmatic lateral surface, inducing weaker pollen competition.

To control for variation in blossom size, which affects seed size (Pélabon *et al*. 2016), we measured the diameter of the blossom peduncle and included it as a covariate in models of seed size (see below). After performing the crosses, the blossoms were bagged and left for ∼5 weeks, the time required for fertilization, seed maturation and explosive dehiscence of the capsules. We monitored the plants daily, so that we could record the maturation time (in days) of the seeds from each blossom. The seeds (*n* = 4068) were counted and individually weighed on a precision balance (precise to 0.1 mg). Abnormal seeds (very small or with empty seed coats) were considered dead and excluded from the seed count.

### Herkogamy, dichogamy and autofertility

We measured ASD (herkogamy) on 1–9 (median = 3) haphazardly chosen blossoms on each plant with digital callipers (precise to 0.01 mm), and computed the mean for each plant. Blossoms were measured on the first day of the bisexual phase (with one male flower open). We conducted an additional experiment to estimate the autofertility of the populations and the level of dichogamy during the development of the blossoms. After removing all open blossoms, three blossoms per plant still in bud were marked with coloured yarn. We then recorded each day the ontogenetic stage of each blossom, scored as either closed (in bud), open (female phase) or open with anthers dehisced (bisexual phase). From this, we could estimate the dichogamy for each blossom (length of the female phase in days). At the end of the bisexual phase, any blossoms that had started to develop fruits were bagged to collect the seeds when mature. Because the plants were grown in a pollinator-free greenhouse, the blossom seed set was used to estimate the autofertility rate of each plant and population.

### Seed germination, seedling survival and early growth

Four sets of seeds, each containing one seed per maternal plant per treatment (*n* = 480 per set), were sown in germination trays covered in plastic to retain moisture. Two sets were sown in normal *Sphagnum*-mixture potting soil (benign environment), and the other two sets were sown in a 1 : 3 mixture of potting soil and perlite (stressful environment). Seeds were assigned random positions in the germination trays. Germination was recorded after 10 days (‘early germination’) and again after 4 weeks (‘late germination/survival’). Seedlings were scored as non-surviving if the seed coat was broken and the seedling had started to germinate, but had later died. At 4 weeks, the above-ground biomass of surviving seedlings was harvested, dried for 72 h at 60 °C and weighed, to obtain dry biomass as a measure of seedling vigour. The decision to terminate the experiment after 4 weeks of seedling growth was based on our previous experience with this species, showing that seedlings that survive until the development of true leaves nearly always survive to flowering and beyond. We, therefore, assumed that survival to 4 weeks represented survival until adulthood.

### Statistical analyses

We estimated inbreeding depression (*δ*) for each population as 1 − *w*_s_/*w*_o_ if *w*_s_ ≤ *w*_o_, and *w*_o_/*w*_s_ − 1 if *w*_s_ > *w*_o_, where *w*_s_ and *w*_o_ are the fitness values of selfed and outbred offspring, respectively. This is identical to the RP index of Ågren and Schemske (1993), except that we estimated inbreeding depression at the population level instead of at the individual level. This index ranges from −1 to 1, with positive values indicating better performance of outbred offspring, and negative values indicating better performance of selfed offspring (Ågren and Schemske 1993, see also Johnston and Schoen 1994). We estimated inbreeding depression for each fitness component separately and for cumulative fitness defined as: number of seeds produced × proportion germinated × proportion surviving × biomass at 4 weeks. Ninety-five per cent confidence intervals (95% CI) of the estimated inbreeding depression were obtained from 1000 non-parametric bootstrap estimates with maternal plants as the unit of resampling.

In order to test for the effect of inbreeding, pollination treatment and population on seed production, we fitted linear mixed-effect models, where cross type (self or cross), pollination treatment (weak or intense pollen competition) and population, as well as their interactions, were treated as fixed effects. Maternal-plant identity was included as a random factor, and peduncle diameter, which correlates with blossom size, was included as a covariate with population-specific slope (i.e. a unique slope was fitted for each population).

Germination success and survival to 4 weeks were analysed using generalized linear mixed-effects models with binomial error distribution and logit link function, where cross type, pollination treatment, population and their interactions were treated as fixed effects; mean seed mass as covariate and germination tray, maternal plant and blossom nested within maternal plant were treated as crossed random effects. In all analyses, the highest ranked models were chosen based on Akaike information criterion (AIC) values of models fitted with maximum likelihood (ML).

Autofertility was estimated as the probability of an ovule being self-fertilized. Probabilities were obtained by inverse logit-transforming (*P* = e*^x^* [1 + e*^x^*]^−1^) the estimates obtained from a generalized linear mixed-effect model with binomial errors and logit link function, where maternal plant and blossom nested within plant were treated as random factors. We estimated autofertility both at the population level (by including population as a fixed factor in the model) and at the plant level (by including maternal plant as a fixed factor).

The relationships between herkogamy, dichogamy and autofertility at the plant level were analysed by fitting an analysis of covariance model where population was included as a categorical variable, and herkogamy and dichogamy as covariates with unique slopes for each population. All continuous variables (autofertility, herkogamy and dichogamy) were standardized to zero mean and unit variance. All analyses were performed in R, version 3.1.1 (R Core Team 2014). Mixed-effects models were fitted using the lme4 package (Bates *et al*. 2014).

## Results

### Herkogamy, dichogamy and autofertility

**Figure 1.**
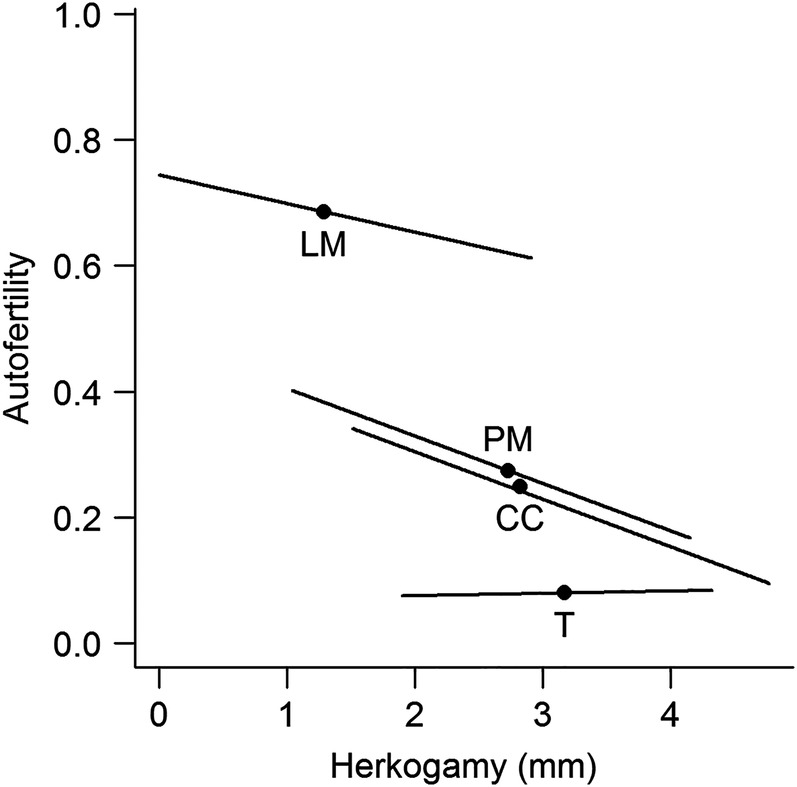
Relationship between ASD (herkogamy) and autofertility, defined as the probability of a seed being produced by self-fertilization, across four populations of *D. scandens*. Population means are shown as black circles, and population-specific slopes are shown over the range of plant-mean ASDs observed in each population. See Table 2 for parameter estimates.

Herkogamy, and to a lesser extent dichogamy, differed among populations (Table 1). Approximately one-third (37.6 %) of the variance in ASD occurred among populations, 16.4 % among plants within populations and the remaining 46 % within plants. For dichogamy only 7.7 % of the observed variance occurred among populations, 20.9 % among plants within populations and the remaining 71.4 % among blossoms within plants. As expected, we observed a negative association between ASD (herkogamy) and autofertility (probability of a seed to be self-fertilized) both among and within populations (Fig. 1), although the relationships between plant-mean herkogamy, dichogamy and autofertility were population specific, and absent in some populations (Table 2). Interestingly, the strongest relationship between individual herkogamy and autofertility occurred for the two populations with intermediate average herkogamy (CC and PM). In the two other populations, either very low (in LM) or large (in T) average herkogamy was associated with little variation in the rate of autofertility. In contrast to herkogamy, dichogamy never detectably affected the rate of autofertility (Table 2).

**Table 2.** Analysis of covariance results for population-specific effects of dichogamy and herkogamy on autofertility in *D. scandens. R* is the Pearson correlation between plant-mean dichogamy and plant-mean herkogamy for each population. All continuous variables were standardized to zero mean and unit variance prior to the analysis, hence the intercepts represent contrasts from the grand-mean-standardized autofertility, and slope estimates represent standardized partial regression coefficients. Autofertility is the probability of a seed being produced by self-fertilization. Model *r*^2^ = 0.70.

Population	*R*	Parameter	Estimate ± SE	*t*	*P*
LM		Intercept	1.00 ± 0.19	5.63	<0.001
	−0.24	Dichogamy	0.19 ± 0.16	1.215	0.23
		Herkogamy	−0.13 ± 0.15	−0.871	0.39
PM		Intercept	−0.14 ± 0.11	−1.257	0.21
	−0.36	Dichogamy	−0.21 ± 0.13	−1.667	0.10
		Herkogamy	−0.39 ± 0.17	−2.284	0.02
CC		Intercept	−0.23 ± 0.13	−1.749	0.08
	−0.21	Dichogamy	−0.08 ± 0.10	−0.842	0.40
		Herkogamy	−0.31 ± 0.14	−2.186	0.03
T		Intercept	−0.83 ± 0.16	−5.358	<0.001
	0.22	Dichogamy	−0.10 ± 0.13	−0.775	0.44
		Herkogamy	0.00 ± 0.18	0.018	0.99

### Seed production

We found no effect of population, cross type (self or cross) or pollination treatment (weak or intense pollen competition) on the number of viable seeds produced (the highest ranked model included only an intercept, see Table 3 for details on model selection). The estimated inbreeding depression for seed production was very low and often negative (fitness inbred > fitness outbred), and the upper limit of the 95 % CI never exceeded *δ* = 0.12 (Table 4). Seed maturation time and mean seed mass differed among populations (Table 5), but again there were no statistically significant effects of cross type or pollination treatment (Table 3, the highest ranked models included population as a fixed effect). Within-blossom proportional variation (CV) in seed mass tended to be greater for selfed seed sets produced under more intense pollen competition in the PM population, but not in other populations (Table 5).

**Table 3.** Model selection results for mixed-effects models testing the effects of population (pop), cross type and pollination treatment (pollen). Peduncle diameter (peduncle) and seed mass are continuous variables (covariates). AIC values were obtained from generalized linear mixed-effect models fitted with ML and with maternal plant treated as a random effect. The highest ranked model for each response variable is presented in bold, together with alternative models that differed by <2 AIC units from the highest ranked model. × indicates an interaction between variables. *k* is the number of parameters in the model. *w* is the Akaike weight, i.e. the relative support for the model given the set of models considered.

Variable	Model parameters	*k*	AIC	ΔAIC	*w*
Maturation time	**pop + peduncle**	**10**	**2272.05**	**0**	**0.25**
	pop + cross type + pop × cross type + peduncle	14	2272.06	0.01	0.24
	pop + cross type + pollen + cross type × pollen + pop × cross type + peduncle	16	2273.58	1.53	0.11
	pop + cross type + peduncle	11	2273.96	1.91	0.09
	pop + pollen + peduncle	11	2274.00	1.95	0.09
	pop + cross type + pollen + pop × cross type + peduncle	15	2274.05	2.00	0.09
Seed set	**constant**	**2**	**1845.98**	**0**	**0.96**
Seed mass	**pop + peduncle**	**10**	**2124.59**	**0**	**0.44**
	pop + pollen + peduncle	11	2126.17	1.58	0.20
	pop + cross type + peduncle	11	2126.24	1.65	0.19
Seed mass CV	**pop + cross type + pollen + peduncle**	**12**	**1632.31**	**0**	**0.22**
	pop + cross type + peduncle	11	1632.39	0.08	0.21
	pop + cross type + pop × cross type + peduncle	14	1634.18	1.87	0.09
	pop + cross type + pollen + pop × pollen + peduncle	15	1634.23	1.92	0.08
	pop + cross type + pollen + cross type × pollen + peduncle	13	1634.26	1.95	0.08
	pop + cross type + pollen + pop × cross type + peduncle	15	1634.28	1.97	0.08
Germination in soil	**cross type + seed mass**	**9**	**624.64**	**0**	**0.29**
	pop + cross type + seed mass	12	625.31	0.67	0.21
Germination in perlite	**pop + cross type + seed mass**	**10**	**531.93**	**0**	**0.14**
	pop + seed mass	9	532.02	0.09	0.14
	cross type + seed mass	8	532.24	0.31	0.12
	seed mass	7	532.38	0.45	0.11
	pop + cross type + pollen + seed mass	11	532.77	0.84	0.09
	pop + pollen + seed mass	10	532.93	1.00	0.09
	cross type + pollen + seed mass	9	533.18	1.25	0.08
	pollen + seed mass	8	533.30	1.37	0.07
Biomass in soil (log)	**pop + pollen + pop × pollen + seed mass**	**14**	**161.82**	**0**	**0.22**
	pop + cross type + pollen + pop × cross type + pop × pollen + seed mass	18	161.96	0.14	0.21
	pop + cross type + pollen + pop × cross type + pop × pollen + cross type × pollen + seed mass	19	163.21	1.39	0.11
	seed mass	7	163.33	1.51	0.11
	pop + cross type + pollen + pop × pollen + seed mass	15	163.78	1.96	0.08
Biomass in perlite (log)	**seed mass**	**7**	**61.53**	**0**	**0.35**
	cross type + seed mass	8	62.73	1.20	0.19
	pollen + seed mass	8	63.52	1.99	0.13

**Table 4.** Summary statistics and estimates of inbreeding depression (*δ*) in four populations of *D. scandens* under intense or weak pollen competition in two soil environments. *w*_s_ and *w*_o_ are the mean (±SE) performance of selfed and outcrossed progeny, respectively, and *δ* is the estimated inbreeding depression (95 % CI). Ninety-five per cent CIs were obtained from 1000 non-parametric bootstrap estimates of *δ* for each fitness components. Significant values are shown in bold. Note that seed production was recorded before the soil and perlite treatments were initiated and these results are, therefore, given only once.

Population	Pollen competition	Seed production (count)			Germination rate			Biomass (mg)		
		*w*_s_	*w*_o_	*δ*	*w*_s_	*w*_o_	*δ*	*w*_s_	*w*_o_	*δ*
Soil										
LM	Intense	8.50 ± 0.18	8.53 ± 0.11	0.00 (−0.04, 0.05)	0.13 ± 0.05	0.28 ± 0.07	**0.51 (0, 0.86)**	34.90 ± 1.84	45.35 ± 2.62	**0.23 (0.11, 0.35)**
	Weak	8.50 ± 0.14	8.33 ± 0.23	−0.02 (−0.08, 0.04)	0.22 ± 0.06	0.21 ± 0.05	−0.02 (−0.53, 0.52)	34.75 ± 1.82	38.00 ± 2.05	0.08 (−0.05, 0.21)
PM	Intense	8.53 ± 0.19	8.43 ± 0.20	−0.01 (−0.07, 0.05)	1.00 ± 0.00	1.00 ± 0.00	0.00 (0, 0)	115.68 ± 8.78	114.77 ± 8.75	−0.01 (−0.19, 0.18)
	Weak	8.70 ± 0.12	8.50 ± 0.14	−0.02 (−0.06, 0.02)	0.98 ± 0.02	0.98 ± 0.02	0.00 (−0.05, 0.05)	138.03 ± 7.32	117.21 ± 7.47	−**0.15 (**−**0.28,** −**0.01)**
CC	Intense	8.17 ± 0.29	8.47 ± 0.27	0.03 (−0.05, 0.12)	0.71 ± 0.08	0.83 ± 0.06	0.14 (−0.09, 0.36)	50.14 ± 1.96	51.49 ± 2.36	0.02 (−0.08, 0.14)
	Weak	8.50 ± 0.26	8.43 ± 0.21	−0.01 (−0.08, 0.07)	0.70 ± 0.08	0.79 ± 0.07	0.12 (−0.13, 0.36)	52.86 ± 2.27	59.38 ± 3.88	0.11 (−0.03, 0.23)
T	Intense	8.53 ± 0.16	8.33 ± 0.22	−0.02 (−0.08, 0.03)	0.88 ± 0.05	0.91 ± 0.04	0.03 (−0.09, 0.17)	60.17 ± 3.97	60.83 ± 3.62	0.01 (−0.14, 0.17)
	Weak	8.80 ± 0.10	8.37 ± 0.36	−0.05 (−0.14, 0.02)	0.92 ± 0.03	0.93 ± 0.03	0.01 (−0.09, 0.10)	56.41 ± 3.10	54.26 ± 3.08	−0.04 (−0.17, 0.10)
Perlite										
LM	Intense				0	0	–	–	–	–
	Weak				0	0	–	–	–	–
PM	Intense				0.94 ± 0.03	0.94 ± 0.03	0.00 (−0.09, 0.09)	94.19 ± 4.74	89.32 ± 5.03	−0.05 (−0.18, 0.09)
	Weak				0.93 ± 0.03	0.96 ± 0.03	0.03 (−0.05, 0.12)	92.26 ± 3.71	84.06 ± 4.60	−0.09 (−0.21, 0.03)
CC	Intense				0.14 ± 0.06	0.18 ± 0.06	0.16 (−0.62, 0.84)	47.46 ± 3.32	45.74 ± 4.67	−0.03 (−0.23, 0.20)
	Weak				0.20 ± 0.06	0.20 ± 0.06	−0.01 (−0.58, 0.58)	52.48 ± 4.66	47.64 ± 3.13	−0.09 (−0.28, 0.11)
T	Intense				0.24 ± 0.06	0.35 ± 0.08	0.30 (−0.23, 0.67)	43.94 ± 6.61	53.71 ± 4.78	0.18 (−0.11, 0.42)
	Weak				0.35 ± 0.08	0.40 ± 0.08	0.12 (−0.39, 0.53)	51.30 ± 3.65	49.57 ± 3.62	−0.03 (−0.20, 0.16)

**Table 5.** Summary statistics for seeds resulting from self- and cross-pollination in *D. scandens* under weak or intense pollen competition.

Population		Treatment			
		Self, weak competition	Self, intense competition	Cross, weak competition	Cross, intense competition
LM	Mean seed mass (mg)	34.78 ± 0.47	34.74 ± 0.47	35.32 ± 0.38	34.91 ± 0.34
	CV seed mass (%)	3.98 ± 0.44	3.78 ± 0.38	2.97 ± 0.20	3.56 ± 0.21
	Maturation time (days)	49.73 ± 1.15	49.47 ± 0.81	48.53 ± 0.65	48.67 ± 0.84
PM	Mean seed mass (mg)	47.49 ± 0.52	46.44 ± 0.64	46.65 ± 0.55	46.99 ± 0.61
	CV seed mass (%)	3.14 ± 0.16	4.21 ± 0.58	3.10 ± 0.29	3.20 ± 0.18
	Maturation time (days)	41.17 ± 0.53	41.57 ± 0.52	40.13 ± 0.49	41.53 ± 0.57
CC	Mean seed mass (mg)	41.19 ± 0.73	41.39 ± 0.82	41.26 ± 0.89	41.20 ± 0.71
	CV seed mass (%)	3.01 ± 0.22	2.69 ± 0.15	2.91 ± 0.24	2.82 ± 0.20
	Maturation time (days)	43.43 ± 0.53	42.83 ± 0.54	43.67 ± 0.50	44.17 ± 0.49
T	Mean seed mass (mg)	42.01 ± 0.49	42.46 ± 0.54	42.14 ± 0.61	42.52 ± 0.53
	CV seed mass (%)	2.54 ± 0.18	2.67 ± 0.23	2.50 ± 0.27	2.49 ± 0.19
	Maturation time (days)	38.80 ± 0.47	38.20 ± 0.43	38.93 ± 0.49	39.13 ± 0.45

### Germination, survival and early growth

At 10 days, the overall germination rate was low and similar in the two soil environments (15.3 and 18.3 % in normal soil and perlite, respectively), and most of the seeds that had germinated at this time were from the PM population. After 4 weeks, the overall germination rate was 71.5 % in normal soil and 36.7 % in perlite, and none of the seeds from the LM population had germinated in perlite (Table 4). Overall, outcrossed seeds were more likely to germinate than selfed seeds both in normal soil and in perlite (Table 3, the highest ranked models included cross type as a fixed factor). Inbreeding depression for germination ranged from −0.02 to 0.51, and was positive (better performance of outcrossed seeds) in most cases (Table 4).

Populations experienced differently the stressful environment imposed. Considering the difference in germination rate of outcrossed seeds in perlite compared with soil as a measure of stress, we noticed that the PM population was not much stressed in perlite, while the CC and T populations were more stressed. For the T population, this was associated with an increase in the observed inbreeding depression for germination in perlite (mean *δ* was 0.02 in soil and 0.21 in perlite, respectively). The LM population failed completely to germinate in perlite, but had a low germinate rate and some inbreeding depression in normal soil.

Nearly all germinated seeds survived until 4 weeks (one seedling died in soil and two in perlite). Therefore, patterns of survival were effectively identical for all populations and treatments, and this fitness component was not analysed further.

In soil, there was a population-specific effect of pollination treatment on seedling biomass (Table 3, the highest ranked model included the population × pollen-competition interaction). Seedling biomass tended to be larger under intense pollen competition in the LM and T populations, but the trend was opposite for the PM and CC populations (Table 4). It should be noted that the apparent effect of seed mass on seedling biomass at 4 weeks (Table 3) can be explained in part by the tendency for heavier seeds (in particular, the seeds from the PM population) to germinate faster, so that these seedlings experienced a longer period of growth before being harvested and weighed. However, this was mostly an among-population phenomenon, and because seed mass was not affected by the treatments in any of the populations, this should not have affected our population-specific conclusions regarding the effect of treatments on early growth.

### Cumulative fitness

**Figure 2.**
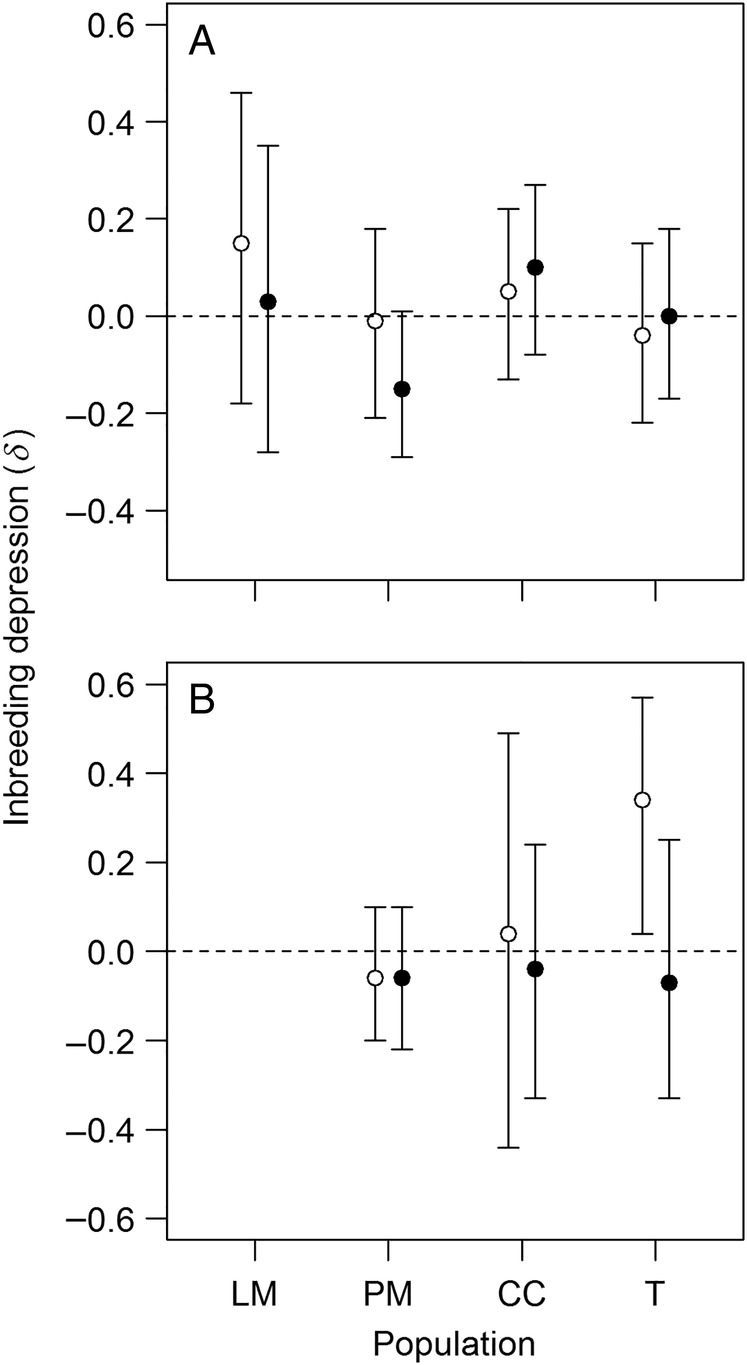
Inbreeding depression (*δ*) for cumulative fitness in four populations of *D. scandens*, in normal soil (A) and perlite (B), under intense (open circles) and weak (filled circles) pollen competition. Error bars indicate 95 % CIs.

Inbreeding depression for cumulative fitness differed somewhat among populations and environments, but was rarely significantly different from zero (Fig. 2). There was some inbreeding depression in the T population in perlite (*δ* = 0.34, 95 % CI = [0.04, 0.57]), but due to the large number of comparisons, this finding should be interpreted with caution. Note also that inbreeding depression in the T population in perlite was greater under intense pollen competition, a result in opposite direction of the predicted pattern. For the other three populations, inbreeding depression for cumulative fitness was never significantly different from zero, as seen from the wide 95 % CIs overlapping zero.

## Discussion

### Floral traits, autofertility and inferred mating history

In plants with mixed mating systems, geographical differences in ‘mating-system traits’, such as herkogamy and dichogamy, are expected to reflect environmental differences including the availability of pollinators and mates, and hence in the frequency of inbreeding events (e.g. Armbruster 1988; Holtsford and Ellstrand 1992; Moeller 2006; Eckert *et al*. 2009). While molecular marker-based methods provide a more direct assessment of mating-system parameters such as outcrossing rates, these estimates tend to be variable among years (e.g. Brunet and Sweet 2006; Eckert *et al*. 2009). Genetically determined differences in herkogamy, like those observed among our study populations, are more likely to reflect long-term trends in selective pressures for selfing or outcrossing. In support of the hypothesized differences in mating histories among our study populations, we found that population-mean autofertility was negatively associated with population-mean ASD (Fig. 1). These results do not necessarily indicate different selfing rates in nature, but suggest that the strength of selection for selfing differs among these populations.

### Patterns of inbreeding depression across life stages and populations

Across the four populations of *D. scandens*, we found some evidence of inbreeding depression only for the probability of seed germination, and this effect depended to some degree on the level of environmental stress experienced by the germinating seeds. Some theory suggests that variation in inbreeding depression due to environmental differences (e.g. following dispersal into a novel habitat, Murren and Dudash 2012; Hereford 2014) may affect the selective advantage of selfing (Cheptou and Mathias 2001). The effect of environmental stress on inbreeding depression has been discussed by several authors (Armbruster and Reed 2005; Cheptou and Donohue 2011; Fox and Reed 2011), and our results further suggest that different populations may experience experimentally induced stress differently. Such differences in stress levels in a common environment are likely to be common when populations originate from different natural environments and have experienced different selective pressures. In our study, the different germination rates of the populations, especially in the dry perlite environment (Table 4), was associated with variation in seed mass. The germination rate was high for the PM population (mean seed mass: 46.9 mg), intermediate for the T and CC populations (41.3 and 42.3 mg, respectively) and low for the LM population (34.9 mg). In the PM population, there was also a trend for heavier seeds to germinate faster (results not shown). Seed mass tends to be greater in dry habitats (Leishman and Westoby 1994), and variation in seed size among populations might, therefore, reflect selection for larger seeds in dryer habitats. This hypothesis could explain the differences in the severity of stress experienced by the different populations in response to the environmental treatment.

Due to the generally weak evidence for inbreeding depression (Table 4, Fig. 2), and the possible confounding effects of stress, we cannot conclude that presumably more inbred populations suffer less from inbreeding depression than do more outbred ones. One possible explanation for this is that all four populations have a history of inbreeding in nature, so that most deleterious alleles have been purged from all of them. Our estimates of autofertility in the greenhouse represent purely autonomous selfing, while realized selfing rates in nature will also depend on geitonogamous (between-blossom) selfing, pollinator-facilitated within-blossom selfing and biparental inbreeding. The pollinators of *D. scandens* (female apid and megachilid bees) routinely visit several nearby blossoms on a single foraging bout, suggesting that geitonogamous selfing and biparental inbreeding might be common (Ø.H. Opedal and E. Albertsen, unpubl. obs.).

Alternatively, inbreeding depression in this system might be expressed at later life stages, such as longevity or lifetime reproductive output. Indeed, some theory predicts (Charlesworth *et al*. 1990), and empirical data support (Husband and Schemske 1996), that purging should act most efficiently on strongly deleterious alleles affecting early-life fitness and that weakly deleterious or late-acting deleterious effects should be much more persistent. This possibility, however, is hard to assess experimentally in perennial species such as *Dalechampia*. In summary, our experimental crosses suggest that inbreeding depression would be a relatively weak force maintaining outcrossing in this system, although late-acting inbreeding depression might be important and warrants further investigation.

### The effect of pollen competition on inbreeding depression

The hypothesis that pollen competition reduces the cost of inbreeding in populations with genetic loads yields three main predictions (Armbruster and Rogers 2004; Lankinen and Armbruster 2007). First, the fitness of self-fertilized offspring should increase under intense pollen competition. Across four populations and four life stages (seed production, germination, survival and early growth), we found no compelling evidence for differences in fitness between self-fertilized offspring produced under intense and weak pollen competition. These results contrast with previous findings in *D. scandens* (Armbruster and Rogers 2004), although these two studies are not directly comparable because they were performed on different populations and used somewhat different pollination treatments (see Pélabon *et al*. 2016 for further discussion). A recent study on the T population (one of the populations used in the present study) also found a similar lack of effects of pollen competition on offspring vigour under outcrossing, and very limited paternal effects on offspring fitness (Pélabon *et al*. 2016).

The second prediction is that inbreeding depression should be reduced under intense pollen competition. We found no significant interaction between cross type (self or cross) and pollination treatment (weak or intense pollen competition) for any fitness component, hence that pollen competition did not detectably affected inbreeding depression. If anything, inbreeding depression tended to be slightly increased under intense pollen competition (Fig. 2).

Because there were very limited differences in inbreeding depression among populations, our data cannot really be used to test the third prediction, that mating history, through its effect on population genetic load, should affect the opportunity for pollen competition to reduce the cost of inbreeding. Taken together, these observations lead us to conclude that the effects of pollen competition on offspring early-life fitness and inbreeding depression in these populations of *D. scandens* were very weak, perhaps because there was very limited genetic load in the four study populations. Studies in additional systems are needed to further evaluate the effect of pollen competition on inbreeding depression.

### Alternative factors promoting outcrossing

If most *D. scandens* populations suffer relatively little from inbreeding depression, it seems likely that other factors contribute to the maintenance of outcrossing. Theoretical work has identified a number of conditions under which continued outcrossing may occur even when inbreeding depression is weak. An example is the existence of functional relationships among fertility components (Johnston *et al*. 2009). The absolute fitness of a plant is determined by the sum of self-fertilized ovules, outcrossed ovules and ovules fertilized through pollen export (male fitness). In their model of mating-system evolution, Johnston *et al*. (2009) found that if positive correlations exist among these fertility components, the evolutionary stable strategy is often mixed mating. Such correlations can arise, for example, if flowers that receive many visits by pollinators also increase their selfing rate through geitonogamous or facilitated selfing, as suggested in *Erythronium grandiflorum* by Holsinger and Thomson (1994). As noted above, geitonogamous and facilitated selfing are likely to be important in *Dalechampia*, and field studies are currently underway to test these expectations.

## Conclusions

### Purging and the stability of mixed mating systems

Across species, inbreeding depression tends to be greater for outcrossing or mixed-mating taxa than for highly selfing taxa (Husband and Schemske 1996; Winn *et al*. 2011, but see Byers and Waller 1999). Within species, studies that have compared populations differing in inferred mating histories have yielded less conclusive results about the effect of purging (see Winn *et al*. 2011 for review; Dart and Eckert 2013). Of the species studied so far, approximately half have shown the predicted trend of decreased inbreeding depression in populations with a history of high selfing rates (studies cited in Winn *et al*. 2011; Dart and Eckert 2013). Conclusions about purging effects in our own study were somewhat complicated by differences in the severity of stress experienced by the different study populations in a common environment. If this has been the case also in other experimental studies of inbreeding depression, it could explain some of the variations in results across different studies. Many studies have compared highly selfing populations or species to more outcrossing ones, and problems associated with differences in stress levels could be especially large in such studies because selfing populations and (sub-)species have been suggested to often occur in more stressful, marginal habitats than more outcrossing populations or congeners (Stebbins 1957). In conclusion, inbreeding depression for early fitness components does not appear to explain the maintenance of mixed mating in *D. scandens*, and further studies should investigate the role of additional factors such as pollinator behaviour and late-acting inbreeding depression.

## Contributions by the Authors

C.P. and Ø.H.O. designed and planned the experiments. Ø.H.O. conducted the experiments, analysed the data and wrote the manuscript with contributions from all the authors.

## Conflict of Interest Statement

None declared.
